# Impact of onychomycosis on the quality of life of patients

**DOI:** 10.18502/cmm.2023.345062.1430

**Published:** 2023-06

**Authors:** Fayrouz Debbagh, Fatima Babokh, Mohamed Sbai, El mostafa El Mezouari, Redouane Moutaj

**Affiliations:** 1 Faculty of Medicine and Pharmacy, Cadi Ayyad University, Marrakesh, Morocco; 2 Parasitology-Mycology Laboratory, Avicenne Military Hospital, Marrakesh, Morocco

**Keywords:** Emotional aspect, Functional aspect, Onychomycosis, Quality of life, Socio-economic aspect

## Abstract

**Background and Purpose:**

Onychomycosis is a very common cosmopolitan onychopathy. It affects the fingers and toes, which are important organs of function and socialization. They can cause physical and psychological discomfort. In this regard, the present study aimed to assess the impact of onychomycosis on the quality of life of patients.

**Materials and Methods:**

This prospective, cross-sectional, observational study was carried out in the parasitology-mycology laboratory of the Avicenne Military Hospital in Marrakesh, Morocco, over 5 months between June and October 2022. The study population was all the patients referred to the laboratory for mycological examination of a nail lesion suspected of onychomycosis of the hands and/or feet.

**Results:**

Onychomycosis was confirmed in 50 patients. Pain, nail thickening, and dyschromia were the most commonly reported symptoms (56%). Onychomycosis had an impact on at least one of the socio-economic, emotional, or functional aspects of the lives of affected patients. More than half (56%) of participants felt embarrassed by the appearance of their nails, 40% hid them, and 28% had a complex about them. The functional aspect was the most bothersome and the time spent on nail care concerned the patients (56%), as well as the discomfort reported when wearing shoes (40%). Women were more worried about the need to hide their nails. A duration of onychomycosis evolution of over 5 years was associated with the highest response rate to the questionnaire (66%).

**Conclusion:**

Although onychomycosis is not a fatal pathology, it significantly reduces the quality of life of affected patients. There is a need to raise the level of awareness of the general population and, above all, of the medical professionals to ensure comprehensive management of onychomycosis.

## Introduction

Onychomycosis or onyxis is defined as a fungal infection of the nail caused by dermatophytes, yeasts, and non-dermatophyte molds [ 1 ]. It is a chronic mycosis affecting the fingernails or toenails. It accounts for 50% of all onychopathies and is becoming increasingly prevalent in the general population [ 2 ]. Although not life-threatening, this common, cosmopolitan pathology may have an influence on the quality of life of the subjects. Onychomycosis is now recognized as a serious health concern as well as an aesthetic issue due to its potential to cause pain, discomfort, and physical deterioration. In addition, the psychological and social limitations caused by onychomycosis can potentially interfere with work and social life. Studies have revealed a psychological and psychosocial impact of up to 92% [ 2 ]. 

This study aimed to evaluate the impact of onychomycosis on the quality of life of affected individuals by identifying the clinical symptoms and socioeconomic, affective, and functional aspects of their daily lives. 

## Materials and Methods

This prospective, cross-sectional, observational study was carried out in the parasitology-mycology laboratory of the Avicenne Military Hospital of Marrakesh, Morocco. This study was conducted over a 5-month period between June and October 2022 and included all patients referred to the laboratory for mycological examination of a nail lesion suspected of onychomycosis of the hands and/or feet who presented clinical signs in the form of thickening, dyschromia, dystrophy, and friability of the nail.

### Data Collection

The patients included in this study underwent mycological examination of their hands and feet after removing their footwear. The evaluated data were collected by the physician on the basis of a 27-item questionnaire, including epidemio-clinical data on age, gender, level of education, location of involvement, duration of symptomatology, and symptoms. The evaluation of the impact on quality of life was organized into three dimensions, namely socio-economic, emotional, and functional. The questions were established based on the experience of the authors and a literature search for similar studies [ 3 ].

### Statistical analysis

All statistical analyses were performed using SPSS software (version 23.0; SPSS, Inc., Chicago, IL, USA) and Microsoft Excel (Microsoft Corporation, Washington, USA). Means and standard deviations for quantitative variables, frequencies, and percentages for quantitative variables were used to describe the data. A probability value (*P*) of less than 0.05 was considered statistically significant.

### Ethics, authorization, and approval

This study was approved by the Institutional Research Ethics Committee of the Faculty of Medicine and Pharmacy of Cadi Ayyad University, Marrakesh, Morocco (N° 545/2022) and was performed in adherence to the Helsinki Declaration. Moreover, informed consent was obtained from all patients before enrollment.

## Results

### Socio-demographic and clinical data

During the study period, onychomycosis was confirmed in 50 patients referred to the parasitology-mycology laboratory. Table 1 summarizes the socio-demographic characteristics of the sample population. The mean age of the patients was 48 years old, with an age range of 15 to 75 years, and there was a male predominance among them (male-to-female ratio of 1.5). The majority of patients included in this study were attending school (84%, n=42), while 34% (n=17) of the subjects had graduated from high school or university. 

**Table 1:** Socio-demographic characteristics of patients with onychomycosis

Gender		N	%
	Female	20	40
	Male	30	60
**Age (years)**	
	<30	6	12
	31-40	8	16
	41-50	14	28
	51-60	10	20
	61-70	10	20
	>71	2	4
**Mean age (years)**		48±14.55	
**Education level**	
	None	8	16
	Elementary school	10	20
	Secondary school	15	30
	University and high school degree	17	34
**Types of onychomycosis**			
	Distolateral subungual onychomycosis	16	32
	Superficial white onychomycosis	3	6
	Proximal onychomycosis	7	14
	Total onychomycodystrophy	24	48
**Location**	
	Fingers	12	24
	Toes	36	72
	Fingers and toes	2	4
**Duration of illness**	
	≤ 2 years	16	32
	2 to 5 years	14	28
	≥ 5 years	20	40
**Predisposing factors**	
	Profession	2	4
	Wearing military boots	18	36
	Diabetes	14	28
	Hammam/Sauna	16	32
	Sport	10	20
	Heat/humidity	16	32
	Manicure and pedicure	4	8

**Figure 1:**
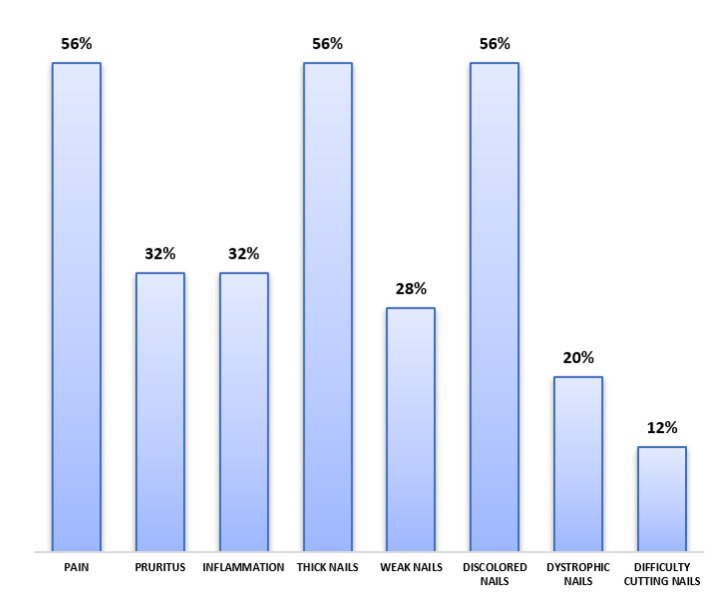
Symptoms reported by patients with onychomycosis

The average duration of symptoms was 6 years, with progression occurring in less than 5 years in 60% of cases (n=30). The most prevalent risk factors in the study population were wearing military boots (36%, n=18), the presence of a heated and humid environment, and hammam bathing (32%, n=16), followed by diabetes (28%, n=14). Toenail onychomycosis was the most common condition (72%, n=36), with onychomycosis-dystrophy lesions predominating. Pain, nail thickening, and dyschromia were the most frequently reported symptoms (56%, n=28), followed by pruritus and nail inflammation (32%, n=16). Figure 1 represents the frequency of symptoms experienced by the study patients.

### Study of the impact of onychomycosis on the quality of life

The evaluation of the quality of life of patients with onychomycosis was based on three primary factors, as outlined in Table 2: socio-economic, emotional, and functional aspects. Data on the socioeconomic effects of onychomycosis revealed that 60% of subjects (n=30) responded positively to at least one question. The primary concern identified was the need to conceal one's nails in public (36%), followed by the dread of spreading the infection to others (32%).

**Table 2:** Responses of patients to the questionnaire on the impact of onychomycosis on the quality of life

Statements		No (n; %)	Yes (n; %)
**Social and economic aspect:**			
	1. I try to hide my nails.	32 (64%)	18 (36%)
	2. My nails look neglected.	40 (80%)	10 (20%)
	3. I am embarrassed when dining out or attending a gathering.	40 (80%)	10 (20%)
	4. People are worried that I will infect them with mycosis.	34 (68%)	16 (32%)
	5. It is really expensive to take care of my nails.	46 (92%)	4 (8%)
**Emotional aspect:**			
	6. I am embarrassed by the appearance of my nails.	22 (44%)	28 (56%)
	7. I have a complex with the appearance of my nails.	36 (72%)	14 (28%)
	8. This problem is affecting my mental health.	46 (92%)	4 (8%)
	9. I worry about having this nail problem for the rest of my life.	46 (92%)	4 (8%)
	10. I worry this might spread.	40 (80%)	10 (20%)
**Functional aspect:**			
	11. I feel uncomfortable wearing my shoes.	30 (60%)	20 (40%)
	12. It is impossible for me to wear any type of shoes.	36 (72%)	14 (28%)
	13. It is difficult to do my leisure and daily activities using my fingers and/or feet.	48 (96%)	2 (4%)
	14. I spend a lot of time cutting my sick nails	22 (44%)	28 (56%)

Emotional impact was noted in 34 patients (64%). More than half (56%, n=28) of the participants were embarrassed by the appearance of their nails, and 28% (n=14) developed a complex over it. However, only 8% (n=4) of those surveyed felt that this issue was detrimental to their mental health and feared it would remain so for the rest of their lives.

The impact of onychomycosis on the functionality of patients was 76% (n=38). The main impact was the time spent caring for the affected nails (56%, n=28), followed by discomfort when wearing shoes (40%, n=20) and the inability to wear any type of shoes (28%, n=14).

Results of the evaluation of responses based on gender are summarized in Table 3. Some aspects of onychomycosis affect females more than males. Females were more concerned about hiding their nails (40%, n=8) and spending a considerable amount of money on nail care (13.3%, n=4), compared to males. The illness had a greater impact on their mental health (13.3%, n=4), and the impossibility of wearing any type of shoes (40%, n=12) with a statistically significant relationship (*P*=0.02).

**Table 3:** Affirmative responses to the Quality-Of-Life questionnaire according to gender

Statements		Male (n, %)	Female (n, %)	*P*-value
**Social and economic aspect:**				
	1. I try to hide my nails.	10 (33.3%)	8 (40%)	0.63
	2. My nails look neglected.	6 (30%)	4 (13.3%)	0.14
	3. I am embarrassed when dining out or attending a gathering.	4 (20%)	6 (20%)	1
	4. People are worried that I will infect them with mycosis.	8 (40%)	8 (26.7%)	0.32
	5. It is really expensive to take care of my nails.	0 (0%)	4 (13.3%)	0.08
**Emotional aspect:**				
	6. I am embarrassed by the appearance of my nails.	12 (60%)	16 (53.3%)	0.64
	7. I have a complex with the appearance of my nails.	6 (30%)	8 (26.7%)	0.79
	8. This problem is affecting my mental health.	0 (0%)	4 (13.3%)	0.58
	9. I worry about having this nail problem for the rest of my life.	2 (10%)	2 (10%)	0.67
	10. I worry this might spread.	6 (30%)	4 (13.3%)	0.14
**Functional aspect:**				
	11. I feel uncomfortable wearing my shoes.	8 (40%)	12 (40%)	1
	12. It is impossible for me to wear any type of shoes.	2 (10%)	12 (40%)	0.02
	13. It is difficult to do my favorite and daily activities using my fingers and/or feet.	2 (10%)	0 (0%)	0.07
	14. I spend a lot of time cutting my sick nails	14 (70%)	14 (46.7%)	0.1

Quality of life of onychomycosis patients depends on the duration of the progression of their symptoms, but with no statistically significant results (Table 4). Therefore, more than half of the patients whose condition had been evolving for more than 5 years had no emotional or socioeconomic impact, at rates of 56.3% and 55%, respectively. Nevertheless, they had more functional concerns (66%), as indicated by at least one affirmative response to a questionary statement.

**Table 4:** Affirmative responses to the Quality-of-Life questionnaire according to the duration of onychomycosis symptoms.

Duration of symptoms	Socio-economic aspect			Emotional aspect			Functional aspect		
	No response	At least one answer	*P*-value	No response	At least one answer	*P*-value	No response	At least one answer	*P*-value
≤ 2 years	30%	70%	0.19	25%	75%	0.31	25%	75%	0.62
2 to 5 years	15%	85%		18.8%	81.2%		41.7%	58.3%	
≥ 5 years	55%	45%		56.3%	43.7%		33.3%	66.6%	

## Discussion

Onychomycosis is a very common pathology, accounting for up to 50% of nail diseases [ 4 ]. It combines various physical symptoms with psychological and social restrictions. This study was conducted to evaluate the influence of onychomycosis on the quality of life of patients in the southeast region of Morocco.

In accordance with other studies [ 5 , 6 ], it was found that onychomycosis was the most prevalent among educated and male subjects within the age range of 41-50 years old. Wearing military or closed shoes was the main risk factor for onychomycosis in this research. This could be explained by the fact that the majority of the participants belonged to military institutions and were frequently required to wear this type of shoes. 

The frequent use of hot baths, hammams, and saunas in the Moroccan culture, as well as the hot and humid atmosphere, have all been identified as factors contributing to the prevalence of onychomycosis. The combination of several risk factors can explain the increase in the prevalence of onychomycosis, including an aging population, the use of immunosuppressive drugs, and the encouragement of the condition by participation in sports and recreational activities [ 7 ]. More than half of the patients (56%) reported pain as the main and first symptom of onychomycosis, which is often correlated with the presence of significant nail thickening. This unpleasant feeling was also observed in other investigations, with episodes of exacerbation often occurring during nail trimming or while wearing tight shoes [ 3 , 8 ]. 

This study demonstrated that onychomycosis affects at least one of the socioeconomic, affective, or functional aspects of the lives of patients. These results are consistent with the conclusions of a number of authors [ 3 , 5 , 6 ] who reported that onychomycosis patients have a lower quality of life. According to Warshaw et al., onychomycosis has comparable symptomatic and functional effects on non-melanodermal skin malignancies and benign lesions, but a greater emotional impact [ 9 ]. 

In the present study, patients complained mainly about the discomfort related to the condition and appearance of their nails, the time spent caring for pathological nails, the discomfort of wearing shoes, and the concern about hiding their nails. Our results revealed the significant impact of onychomycosis on the functional aspect, with a 76% positive response rate. In fact, fingers and toes are used for basic physical activities (e.g., walking and manual dexterity) as well as work and social activities on a daily basis. In the case of onychomycosis, their functions may be compromised. 

Similar to Drake et al. [ 3 ], 40% of our patients experienced discomfort when wearing shoes. However, females were statistically more affected by the inability to wear any type of footwear (*P*=0.02), likely due to their interest in wearing tight heels, which are uncomfortable for the foot regardless of any pathology. 

One of the main socio-economic difficulties reported by the patients was the concern about hiding pathological nails (36%), and the fear of transmitting the disease to others (37%). Similar findings were reported in other investigations. According to Szepietowski et al. [ 5 ], patients complained of embarrassment about the condition of their nails and concern about spreading the infection. Elewski et al. [ 8 ] noted a negative psychological and psychosocial impact of onychomycosis in all examined patients. According to them, subjects suffered mainly from social stigmatization of their condition, which made social acceptance and desirability difficult. This is due to falsehoods and misunderstandings, which must be refuted through educational campaigns in order to increase understanding and awareness of the illness [ 10 ]. 

It is also essential to consider the financial concerns that patients may have. The worry about spending more, stated mainly by women in this population, is especially troubling for those with financial issues. Therefore, the diagnosis of onychomycosis can become a source of financial burden and increase the concerns and anxiety of the patients [ 4 ].

Evaluation of the impact of onychomycosis on the quality of life of the patients revealed that 82.2% of those with a disease duration of 2 to 5 years have at least one emotional concern. Discomfort with the appearance of nails was the most impacted element (53.3%) among the study population. Clean, healthy, and well-maintained nails are indicative of physical wellness and health. The damages caused by onychomycosis can cause self-esteem and confidence issues, as well as a decline in body image and self-perception. 

The present study revealed that the impact of onychomycosis on the quality of life of patients was mainly determined by the duration of disease progression and gender. Onychomycosis had a significantly greater socioeconomic and psychological impact on patients with a disease duration of 2-5 years, compared to those with a disease duration of less than 2 years. Over 5 years of onychomycosis progression was associated with a higher response rate (66%) to the questionnaire. These results are consistent with those of other studies [ 3 , 5 , 6 ] which reported that the duration and severity of onychomycosis were significantly related to the quality of life of patients. However, Tabolli et al. [ 10 ] did not identify a correlation in this sense. 

The perception of the impact of onychomycosis on the quality of life of affected individuals varies between countries due to cultural disparities. Drake et al. [ 3 ] concluded in an international study that onychomycosis impacted the quality of life of some populations more than others. In fact, German and American patients felt more severely impacted by onychomycosis than Italians [ 3 ]. 

Nevertheless, it should be noted that this study had some limitations. First, the limited sample size did not allow statistically significant results or a comparison between two groups of patients with and without disease. Second, the used questionnaire was not specific to fingers or toes, making it impossible to draw conclusions regarding the impact based on the location of onychomycosis. The difference in perception of the pathology across the regions of Morocco was not assessed. It is recommended to conduct additional research in order to capitalize on the disparities that would likely exist. In addition, the impact of the treatment on the quality of life of onychomycosis patients must be evaluated for a more global estimation of this parameter. Finally, the financial impact was underestimated in the absence of real data on the economic cost of this disease.

## Conclusion

Although onychomycosis is not a fatal disease, it significantly reduces the quality of life of those affected. There is a need to increase the level of promotion of public knowledge regarding the prevention of fungal infection and non-transmission and the availability of effective curative treatments. Patients must be aware of the importance of proper medical consultation in the diagnosis of onychomycosis at the first signs of the condition. Health professionals must also be conscious of the varying effects of onychomycosis on life quality and communicate information about this highly stigmatized illness. Serious consideration should be given to these patients to support and reassure them positively throughout the therapeutic process.
